# Enhanced Biocompatibility and Osteogenic Activity of Marine-Plankton-Derived Whitlockite Bone Granules through Bone Morphogenetic Protein 2 Incorporation

**DOI:** 10.3390/bioengineering9080399

**Published:** 2022-08-17

**Authors:** Ji Won Baek, Ki Su Kim, Ho Park, Nak Gyu Park, Beom-Su Kim

**Affiliations:** 1 Department of R&BD, Cellco Inc., 208, Venture Startup Center, Jeonju University, 303, Cheonjam-ro, Wansan-gu, Jeonju-si 55069, Korea; 2 Department of Clinical Laboratory Science, Wonkwang Health Science University, 514, Iksan-daero, Iksan-si 54538, Korea; 3 Division of Mechanical Design Engineering, Jeonbuk National University, Jeonju-si 54896, Korea; 4 Carbon Nano Convergence Tech Center, Jeonbuk National University, Jeonju-si 54896, Korea

**Keywords:** whitlockite, marine plankton, bone graft, bone morphogenetic protein 2, bone regeneration

## Abstract

Whitlockite (WH) is a calcium-phosphate-based Mg-containing ceramic with good mechanical properties, rapid resorption, and good osteogenicity. Recently, we successfully synthesized highly porous WH granules using a marine plankton exoskeleton (MP-WH). In the present study, we improved the osteoinductive activity of MP-WH granules by bone morphogenetic protein2 (BMP2) (MP-WH/BMP2). The surface morphology and composition of the fabricated MP-WH/BMP2 granules were characterized using scanning electron microscopy (SEM), X-ray diffraction, and Fourier transform infrared (FT-IR) spectroscopy. The biocompatibility and osteogenic effects were evaluated using human mesenchymal stem cells (hMSCs). BMP2 was absorbed on the surfaces of the MP-WH/BMP2 granules. Immobilized BMP2 was released at a moderate rate over 30 days. hMSCs seeded on MP-WH/BMP2 granules became biocompatible, with a better proliferation and adhesion for MP-WH/BMP2, compared with MP-WH. Bone-specific markers Runx2, type I collagen, osteocalcin, and osteopontin were significantly upregulated following BMP2 incorporation. Similar observations were made regarding the alkaline phosphatase activity. This study suggests that BMP2 incorporation improves the osteoinductive activity of marine-plankton-derived WH granules for bone tissue repair.

## 1. Introduction

Bone defects can arise owing to several conditions, including trauma, tumors, and bone diseases. However, critical-size bone defects cannot heal independently. Therefore, artificial bone replacement has the potential to repair bone defects [1,2]. Autologous bone is the gold standard for graft materials because it supports bone conduction, osteoinduction, and osteogenesis. However, autologous bone transplantation often suffers from several complications, such as morbidity and quantity limitations at the donor site [3]. To overcome these disadvantages, calcium phosphate ceramics are widely used as bone substitutes, because they are similar to bones. Whitlockite (WH) is the second most abundant bone component. It is a calcium phosphate ceramic containing the mineral magnesium, which accounts for 25–35% of the mineral fraction of human bones [4]. WH has an excellent compressive strength, high absorption rate, and excellent bone formation properties compared with other ceramics [4]. In particular, the magnesium ions released from WH promote osteoblast differentiation during the early stages of bone formation [5]. Although several studies have reported that metal ions (such as magnesium ions) promote osteoinductivity [6], WH still exhibits insufficient osteoinductivity, and a strategy to compensate for this is needed. 

To promote osteoinductivity, several growth factors such as bone morphogenetic protein (BMP), fibroblast growth factor (FGF), and vascular endothelial growth factor (VEGF) have been used in bone tissue engineering [7]. Although many growth factors have been shown to be promising for bone regeneration and repair, BMP is the most studied osteoinducing factor [8]. BMP2 belongs to the superfamily of tumor growth factor β (TGF-β) proteins, and has been shown to regulate osteoblast differentiation. In addition, BMP2 is a promising therapeutic agent that promotes bone regeneration when delivered topically with bone substitutes. For example, Notodihardjo et al. [9] reported that a BMP2/hydroxyapatite (HA) composite exhibited the highest level of bone induction, compared with the group for which only HA was used. In addition, Jang et al. [10] showed that bone healing was significantly improved for the BMP2 and biphasic calcium phosphate combination group, at either 2 or 8 weeks, in a rat calvarial defect model, compared with the biphasic calcium phosphate implanted group. These reports indicate that locally delivered BMP2, combined with bone substitutes, can synergistically enhance bone formation. 

Recently, we successfully synthesized highly porous WH granules using a marine plankton (MP) exoskeleton of foraminifera. Foraminifera produce shells that can have multiple chambers, and the structure of these shells is unique. In particular, the exoskeleton of foraminifera forms a complex porous network, and this structure is highly suitable as a porous feature for bone regeneration.

Therefore, in this study, we aimed to prepare BMP2-incorporating marine-plankton-derived WH (MP-WH) granules for improving the osteoinduction activity. The morphological and chemical properties of the synthesized BMP2 incorporating MP-WH (MP-WH/BMP2) granules were characterized. The in vitro biocompatibility was evaluated using human mesenchymal stem cells (hMSCs). The in vitro osteoinduction activity was determined by real-time polymerase chain reaction (RT-PCR) and alkaline phosphatase activity. 

## 2. Materials and Methods

### 2.1. Preparation of Porous WH Granules

The foraminiferous exoskeleton (*Baculologypsina sphaerulata*, Okinawa, Japan) was obtained from the market. To synthesize the WH granules, a hydrothermal transformation was performed using previously established methods [11]. Briefly, a (Ca + Mg):P molar ratio was achieved by immersing the samples in an aqueous (NH_4_)H_2_PO_4_ solution added to the bone. The samples were then placed in a Teflon-lined stainless-steel pressure vessel and heated at 200 °C for 24 h. The transformed samples were washed with boiling water and dried at 60 °C. The samples were then calcined at 900 °C for 6 h. Then, the WH granules were chopped using a lancet, and granules with sizes in the ~300–700 μm range were isolated using a stainless-steel sieve.

### 2.2. Adsorption of BMP2 onto WH Granules

For this study, rhBMP2 was purchased from Sigma-Aldrich (St. Louis, MO, USA). Briefly, the MP-WH granule sample (200 mg) was immersed in 200 μl of the BMP2 solution (100 μg/mL) and incubated for 60 min at 4 °C. The resultant slurry was then freeze-dried for 24 h to obtain BMP2-incorporating MP-WH (MP-WH/BMP2) granules. 

### 2.3. Physicochemical Analysis

To characterize the composition of the transformed foraminifera-derived WH granules, X-ray diffraction analysis (XRD; D8, Bruker AXS, Karlsruhe, Germany) was performed using CuKα radiation at 0.02 °/min (2θ), a scanning speed of 50 kV, and a 30 mÅ in the 10–80° range. The microstructure and surface morphology of the MP-WH granules were observed under vacuum conditions using scanning electron microscopy (SEM; EM-30, COXEM, Daejeon, Korea). BMP2 incorporation was performed using thermogravimetric analysis (TGA) and a Fourier transform infrared (FT-IR) spectrometer (Frontier, PerkinElmer, Waltham, MA, USA), in the 400–4000 cm^−1^ range. 

### 2.4. Compressive Strength and Porosity

The compressive strength of the granules was measured according to ISO 13175-3. After filling the inner region of a ring-shaped mold (diameter, 10 ± 1 mm; thickness, 2 ± 0.2 mm), the mold was carefully removed, and the cross-head speed of the universal testing machine was set to 0.5 ± 0.1 mm/min. A load was applied until the granules broke down. Granular porosity was assessed using a mercury intrusion porosimeter. The pore volume was determined from the amount of mercury that infiltrated at a known pressure. AutoPore IV 9500 (OakRidge, TN, USA) was used within a measurable pore diameter range of 0.006–800 µm. Samples weighing approximately 0.1–0.15 g were used.

### 2.5. Cumulative Release of BMP2

The release profiles of BMP2 were determined using an enzyme-linked immunosorbent assay (ELISA). The BMP2-incorporating MP-WH sample was immersed in 1.5 mL of phosphate-buffered saline (PBS) (pH 7.4). The tubes were then incubated at 37 °C. At various time points, the supernatant was collected and fresh PBS was added. The BMP2 concentration was determined using an ELISA kit (R&D Systems, Minneapolis, MN, USA). 

### 2.6. hMSC Culture 

The biocompatibility and osteoinduction activity of the synthesized MP-WH/BMP2 granules were evaluated using hMSCs purchased from ATCC (Manassas, VA, USA). The cells were cultured in α-MEM (Gibco BRL, Gaithersburg, MD, USA) containing 10% fetal bovine serum (FBS; Gibco BRL) and 1% penicillin/streptomycin, at 37 °C, in an atmosphere containing 5% CO_2_ at 100% humidity. The cultured hMSCs were passaged 3–6 times. 

### 2.7. Cell Culture on MP-WH /BMP2 Granules

MP-WH and MP-WH/BMP2 granules were sterilized using gaseous ethylene oxide. For the in vitro cell culture, 20 mg of granules was added per a 96-well plate, and hMSCs were seeded onto the granules. After 4 h of incubation to allow for cell adhesion, the granules were washed using a cell culture medium to remove non-adherent hMSCs, and then were transferred into new cell culture plates.

### 2.8. Proliferation of hMSCs Cultured on MP-WH/BMP2 Granules

To assess cell proliferation, an MTS assay was performed at predetermined time points (1, 5, 10, and 15 days). Briefly, at each time point after culturing, the cell growth medium was removed, following which the cell-cultured granules were rinsed using PBS. A fresh culture medium (200 μL) and MTS reagent were mixed and added to each well. After incubation at 37 °C for 2 h, the resultant supernatant was collected and the absorbance was measured at 450 nm using a microplate reader (SpectraMAX M3; Molecular Devices, Sunnyvale, CA, USA). To confirm cell proliferation, the total amount of deoxyribonucleic acid (DNA) was quantified using the PicoGreen assay (Molecular Probes, Eugene, OR, USA) after five days of cultivation. Briefly, the total amount of DNA was extracted from the hMSC-cultured MP-WH and MP-WH/BMP2 granules. The DNA solution (100 µL) was mixed with a DNA-binding fluorescent dye solution (0.5 µL PicoGreen reagent), and the fluorescence intensity was measured at excitation (490 nm) and emission (520 nm) wavelengths.

### 2.9. Cell Viability and Cytotoxicity 

To evaluate the cell cytotoxicity, viability/cytotoxicity kits (Invitrogen, Carlsbad, CA, USA) were used. Briefly, after 1, 2, and 5 days, the cells cultured on the MP-WH and MP-WH/BMP2 granules were rinsed with PBS. The cells were stained using the kit solutions (Calcein AM and EthD-1) and were observed under an inverted fluorescence microscope (DM IL LED Fluo; Leica Microsystems, Wentzler, Germany).

### 2.10. Observations of Cellular Adhesion to MP-WH and MP-WH/BMP2 Granules

The cellular adhesion of hMSCs to the MP-WH and MP-WH/BMP2 granules was observed using SEM. The hMSCs were cultured for five days, and the cell-cultured granules were washed with PBS. For fixation, the cells were treated with 2.5% glutaraldehyde for 1 h. Then, the samples were post-fixed with 2% osmium tetroxide (OsO_4_; Sigma-Aldrich, St. Louis, MO, USA) for 30 min. For dehydration, 25%, 50%, 75%, 90%, and 100% ethanol were used. After dehydration, the samples were coated with platinum and cell adhesion was observed using SEM (EM-30).

### 2.11. Alkaline Phosphatase Activity

The osteoblast differentiation activity of the hMSCs cultured on the synthesized MP-WH and MP-WH/BMP2 granules was determined using an alkaline phosphatase (ALP) activity assay. Briefly, the hMSCs were seeded on the granules and were cultured for seven days. To obtain the total protein, 1% Triton X-100/PBS solution was added and sonicated for 10 min in an icebox. To remove the granule debris, the treated samples were then centrifuged at 12,000 rpm at 4 °C, and the resultant supernatant was collected. As described previously [12], *p*-nitrophenylphosphate (*p*-NPP) was used as a substrate, and the measured ALP activity was normalized to the total protein content. 

### 2.12. RT-PCR for Osteoblast-Related Gene Expression

To determine the osteogenic differentiation, several osteogenesis-related genes were assessed using quantitative RT-PCR (qRT-PCR). Briefly, the hMSCs were seeded on the MP-WH and MP-WH/BMP2 granules and cultured for seven days. Next, the total messenger ribonucleic acid (mRNA) was extracted, and complementary DNA (cDNA) was synthesized using reverse transcriptase. Next, qRT-PCR was performed using probe sets that specifically targeted runt-related transcription factor 2 (Runx2), collagen type I, osteocalcin (OCN), and osteopontin (OPN; Applied Biosystems, Carlsbad, CA, USA). Finally, the 18S ribosomal ribonucleic acid (rRNA) gene was used as an internal standard, and the relative expression was normalized to the MP-WH granule. 

### 2.13. Immunocytochemistry 

After five days of cultivation, hMSCs cultured on the MP-WH and MP-WH/BMP2 granules were rinsed with PBS and treated with 4% paraformaldehyde. Anti-pSmad 1/5/8 rabbit IgG (Abcam, Cambridge, UK) and Alexa Fluor 594 donkey anti-rabbit IgG (Invitrogen, Carlsbad, CA, USA) were used to determine the pSmad expression. The nuclei were stained with a 4′,6-diamidino-2-phenylindole (DAPI) dye solution. The samples were observed using a fluorescence microscope (DM IL LED Fluo, Leica, Microsystems, Wetzlar, Germany).

### 2.14. Statistical Analysis

All experiments were performed in triplicate for each condition and were repeated at least twice. Values are reported as the mean ± standard deviation (SD). Statistical analysis was performed using one-way analysis of variance, and post hoc comparisons were performed using Tukey’s correction using GraphPad Prism software (San Diego, CA, USA). The significance level was set at *p* < 0.05.

## 3. Results

### 3.1. Preparation of WH Bone Granules from Marine Plankton

**Figure 1:**
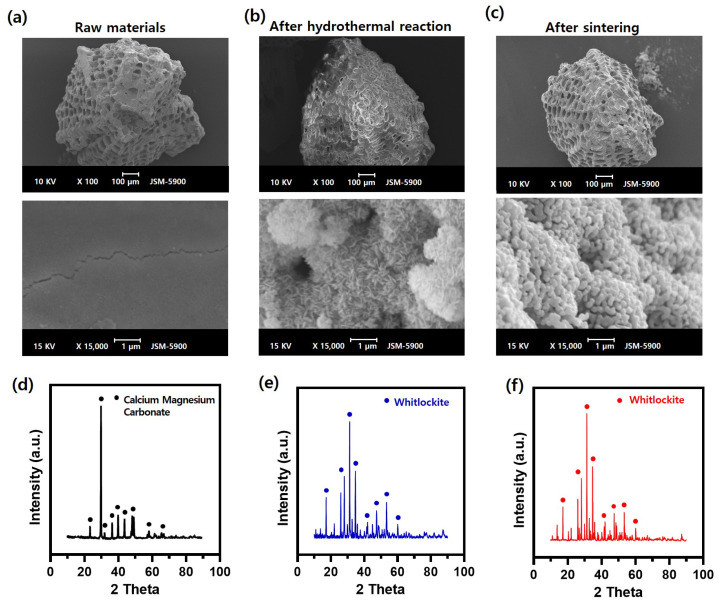
Surface morphology of marine-plankton-derived WH bone granules: (**a**) SEM images of the raw material, (**b**) material after the hydrothermal reaction, and (**c**) material after the sintering process. Characterization of the granules according to the process using XRD for the (**d**) raw material, (**e**) material after the hydrothermal reaction, and (**f**) material after the sintering process.

In this study, a marine plankton skeleton was used for synthesizing WH bone granules. The plankton skeleton used as a raw material had a unique microporous structure, and its amplified surface morphology image revealed a smooth surface. XRD analysis confirmed that the synthesized WH bone granules were composed of calcium magnesium carbonate (Figure 1a,d). The sample in which the hydrothermal synthesis reaction was completed using the raw material had a typical needle-like structure formed by synthetically grown WH on the surface (Figure 1b). After the hydrothermal reaction, the XRD characterization results revealed that the raw material was converted to WH (Figure 1e). In addition, the sample after the sintering process (the last step in the synthesis process) exhibited the formation of agglomerated particles (Figure 1c), and its chemical composition was consistent with the WH peak observed in the XRD analysis (Figure 1e).

### 3.2. BMP2-Incorporating WH Bone Granules 

**Figure 2:**
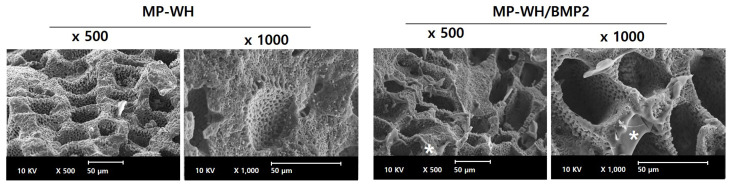
SEM images of BMP2-incorporating marine-plankton-derived WH granules. The images reveal that BMP2 irregularly covers the granules’ surface. Asterisks indicate the fraction of incorporated BMP2.

BMP2 was incorporated into MP-WH bone granules by absorbing the BMP2 solution, and a lyophilizing method was used. There was no significant change in the porous structure following BMP2 incorporation. In addition, the surface of the bone graft material to which the BMP2 was adsorbed did not change significantly, but the agglomerated lyophilized pellets were rarely observed on the surface (Figure 2).

### 3.3. Characterization of BMP2-Incorporating MP-WH Granules 

**Figure 3:**
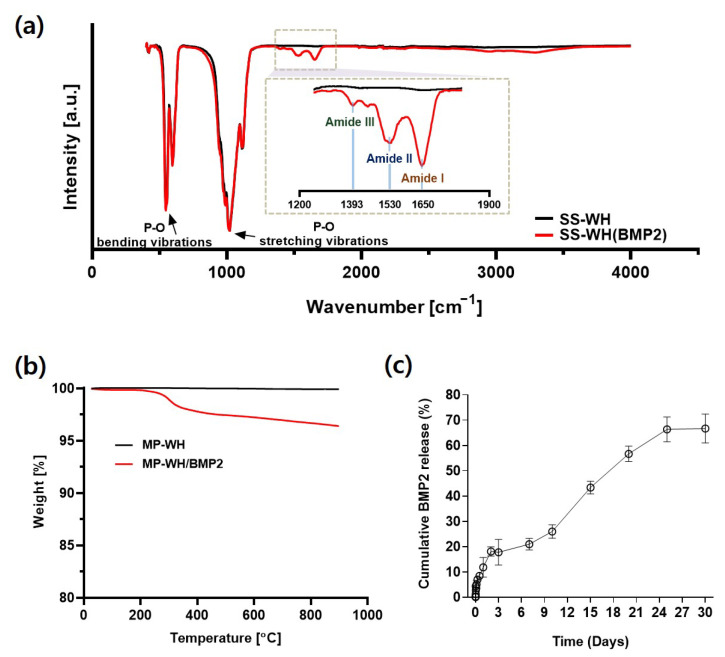
Physicochemical characterization of BMP2-coated MP-WH granules: (**a**) FT-IR spectra of BMP2-incorporating MP-WH granules, (**b**) TGA of BMP2-coated MP-WH granules, and (**c**) cumulative release of BMP2 from MP-WH/BMP2 granules for 30 days. The supernatant is collected at specific time points and the concentration is detected using the ELISA method. The reported values are means ± SD.

BMP2 incorporation into MP-WH granules was characterized using FT-IR spectroscopy and TGA. In both normal MP-WH granules and MP-WH/BMP2 granules, peaks corresponding to phosphate were observed at 1019 cm^−1^ (asymmetric P-O stretching vibrations, v3), 950 cm^−1^ (symmetric P-O stretching vibrations, v1), 560 cm^−1^, and 420 cm^−1^ (bending vibrations of P-O bonds, v4 and vz). On the other hand, the difference between the peaks in the spectra of the normal MP-WH granules and MP-WH/BMP2 granules was evident in the 1300–1700 cm^−1^ range. Only in the MP-WH/BMP2 granular spectrum did the peaks at 1650, 1530, and 1393 cm^−1^ appear, corresponding to the specific peaks of amide I, amide II, and amide III of the protein, respectively (Figure 3a). Furthermore, BMP2 coating was confirmed by TGA. In the MP-WH sample, there was no change in weight % as the temperature increased. However, the weight % decreased sharply within the 200–400 °C range and decreased gently in the 400–900 °C range in the MP-WH/BMP2. As the organic matter was sintered from the granules, the weight curve decreased. The FT-IR and TGA results suggest the successful coating of BMP2 on the MP-WHG granules (Figure 3b). Next, the release pattern of the BMP2 protein from the synthesized bone granules was evaluated. Under in vitro conditions, BMP2 exhibited a rapid release pattern up to the first three days. Subsequently, the release proceeded slowly, and the release of approximately 70% of BMP2 continued until 30 days (Figure 3c).

### 3.4. Compressive Strength and Porosity of MP-WH/BMP2 Granules

**Figure 4:**
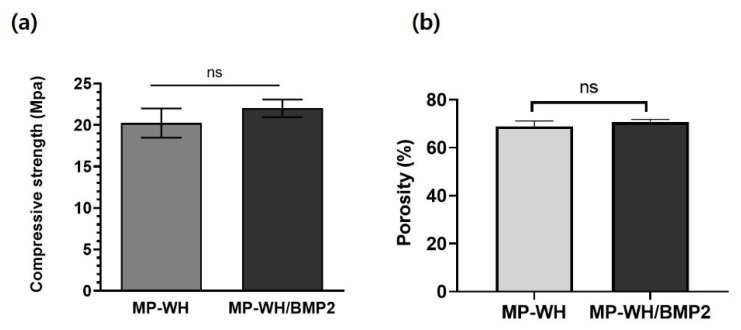
Measurements of the granules’ mechanical strength and porosity: (**a**) compressive strength and (**b**) porosity of the synthesized MP-WH and MP-WH/BMP2 granules. Data are means ± standard deviation (SD) over three samples. “ns” indicates significant differences, compared with the uncoated three-dimensional scaffolds (*p* < 0.01).

We evaluated the compressive strength and porosity following the incorporation of BMP2. The granules before the BMP2 coating exhibited a compressive strength of 20.5 ± 2.3 MPa. After the BMP2 coating, the coated MP-WH/BMP2 granules exhibited a slightly higher compressive strength of 21.9 ± 1.6 MPa, but the difference was not statistically significant (Figure 4a). In addition, the MP-WH granules exhibited a porosity of 68.5 ± 2.2%, while the MP-WH/BMP2 granules exhibited a porosity of 69.2 ± 1.6% (Figure 4b). These results indicate that the incorporation of BMP2 through adsorption and lyophilization did not affect the total porosity and compressive strength of the bone graft material derived from marine plankton.

### 3.5. Cell Proliferation on MP-WH/BMP2 Granules

**Figure 5:**
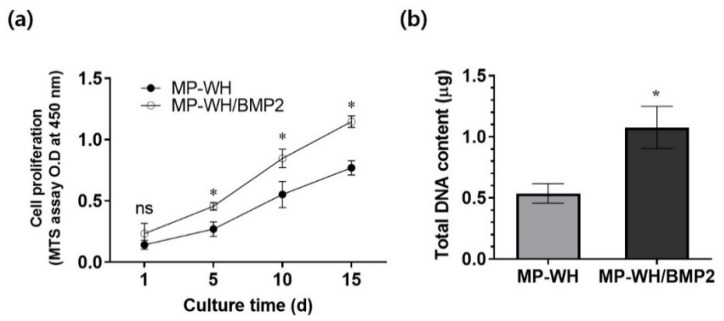
Evaluation of hMSCs proliferation following BMP2 incorporation: (**a**) cell proliferation evaluated using the MTS assay at 1, 5, 10, and 15 days; (**b**) to confirm the cell proliferation, a DNA content assay is performed five days after the seeding. The presented data are means ± standard deviation (SD) of three samples. * *p* < 0.05 indicates significant differences with respect to the uncoated scaffolds. ‘ns’ indicates not significant.

The proliferation of cells growing on the synthesized MP-WH and MP-WH/BMP2 granules was evaluated using the MTS assay. hMSCs grew well over time, and their proliferation was significantly higher on the MP-WH/BMP2 granules compared with the uncoated granules (MP-WH). In addition, until day 15, the proliferation of the cells cultured on the MP-WH/BMP2 granules was higher than that of the cells cultured on the MP-WH granules (Figure 5a). A DNA content assay was performed to confirm the cell proliferation activity. The results of this analysis (Figure 5b) showed that the density of the cells that were cultured on the MP-WH/BMP2 granules (1.19 ± 0.22 μg) was higher than that of the cells that were cultured on the MP-WH granules (0.51 ± 0.11 μg). The results of the total DNA content analysis were consistent with those of the MTS assay.

### 3.6. Cytotoxicity of MP-WH/BMP2 Granules

**Figure 6:**
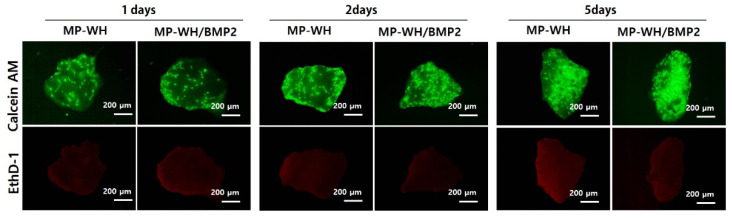
Cytotoxicity of MP-WH and MP-WH/BMP2 granules. The cell viability/cytotoxicity of MSCs is determined using live/dead fluorescence staining with Calcein AM and EthD-1 after 1, 2, and 5 days of cultivation. Calcein-AM stained healthy live cells are shown in green, while EthD-1 stained dead and unhealthy cells are shown in red.

Cytotoxicity testing is the most fundamental step in the development of biomaterials. We performed live/dead fluorescence staining to evaluate the toxicity of the developed marine-plankton-derived WH bone graft material, as well as the properties of the graft material after BMP2 coating. Our live/dead fluorescence staining results showed that mesenchymal stem cells growing on the synthesized MP-WH granules were stained with calcein-AM and exhibited green fluorescence. In contrast, dead cells stained with red dots for EthD-1 were not observed. In addition, cells cultured on the MP-WH/BMP2 granules also attached well to the granule surface and grew, and cells that died from EthD-1 were not observed. The results of this live/dead fluorescence staining experiment suggest that MP-WH/BMP2 is a safe biomaterial that does not exhibit cytotoxicity (Figure 6). In addition, the density of the growing cells attached to the different granule-type surfaces was higher for the MP-WH/BMP2 granules than for the MP-WH granules, consistent with previous MTS cell proliferation results.

### 3.7. Cellular Adhesion to MP-WH/BMP2 Granules

**Figure 7:**
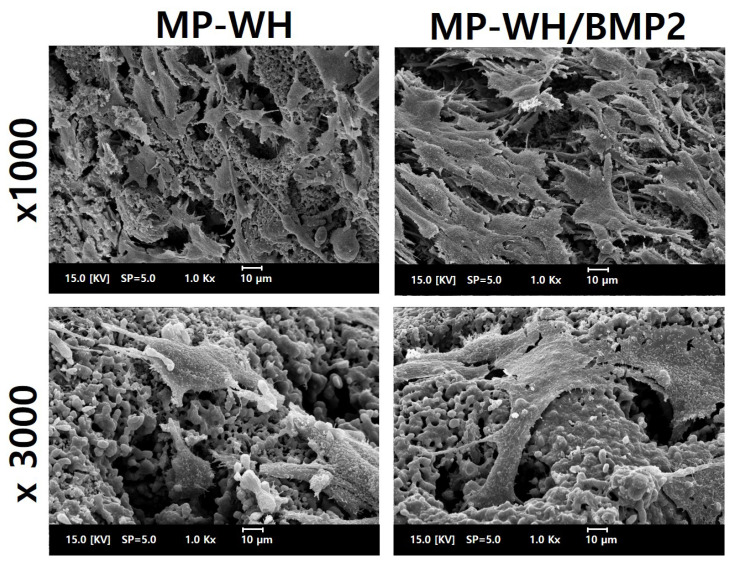
SEM images of cellular adhesion and growth. Cells are cultured on the surfaces of the synthesized MP-WH and MP-WH/BMP2 granules, and their morphologies are observed using SEM after three days in culture.

SEM analysis was performed to observe the adhesion of cells to the surface of the bone graft material. On the MP-WH surface, hMSCs grew and adhered, while maintaining their typical fibroblastic morphology. In addition, under high magnification, these cells were observed to grow and enter the pore structure of the MP-WH granules. The cells that were cultured on the MP-WH/BMP2 granules grew well, maintaining their typical fibroblast morphology. High-magnification observations revealed that the cells flexibly bent inward along the pore structure and attached. However, comparing these two observations, the cells grown on the MP-WH/BMP2 granules had a higher cell density than those grown on the MP-WH granules, and growth of overlapping cell layers was observed (Figure 7).

### 3.8. Osteogenic Activity of MP-WH/BMP2 Granules

**Figure 8:**
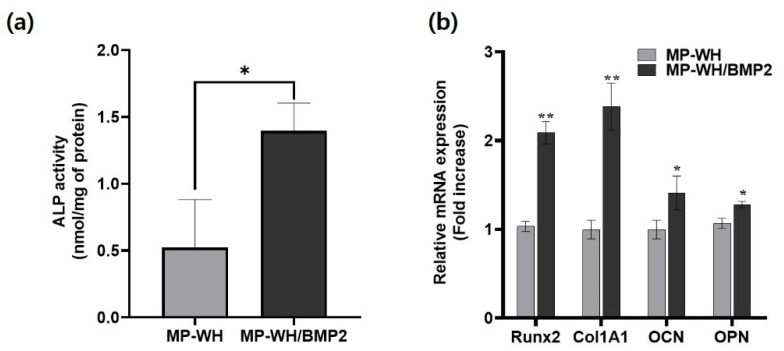
Effects of the BMP2 incorporation into MP-WH on osteoblast differentiation. hMSCs are cultured on MP-WH and MP-WH/BMP2 granules, and osteoblast differentiation are determined by (**a**) the ALP activity assay and (**b**) RT-PCR for specific osteoblast marker genes. Data are means ± standard deviation (SD). * *p* < 0.05 and ** *p* < 0.01 indicate significant differences of MP-WH/BMP2 from MP-WH.

To evaluate the effect of BMP2 incorporation on osteoblast differentiation, the ALP activity and osteoblast differentiation marker gene expression were evaluated using RT-PCR. After a 7-day-long cultivation period, the ALP activity for the MP-WH/BMP2 grown cells was significantly (~3 fold) higher than that for the MP-WH-grown cells (Figure 8a). Furthermore, qRT-PCR analysis revealed the mRNA levels of Runx2 (~2.1 fold), Col1A1 (~1.4 fold), OCN (~1.2 fold), and OPN (~1.2 fold); the mRNA levels were significantly higher for the MP-WH/BMP2-cultured cells (Figure 8b).

### 3.9. Phosphorylation of Smad 1/5/8 by MP-WH/BMP2

**Figure 9:**
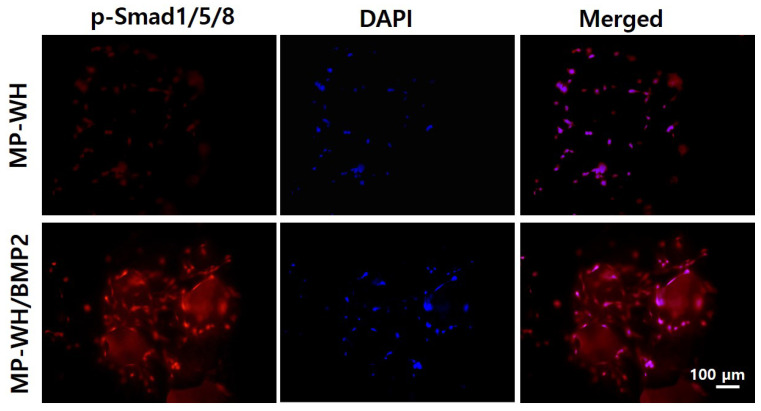
Activation of MP-WH/BMP2-induced pSmad 1/5/8 signaling: hMSCs cultured on MP-WH and MP-WH/BMP2 granules, and Smad 1/5/8 phosphorylation detected by immunofluorescence staining via DAPI (blue) and anti-pSmad 1/5/8 (red).

To evaluate the association between the MP-WH/BMP2-induced osteoblast differentiation and pSmad 1/5/8 signaling, immunocytochemistry staining was performed. Basal level phosphorylation of Smad 1/5/8 was observed for hMSCs cultured on MP-WH granules. The phosphorylation level of pSmad 1/5/8 in adherent cells on the surface of each granule was higher for MP-WH/BMP2 than for MP-WH (Figure 9).

## 4. Discussion

For tissue regeneration, stem cells are considered an attractive alternative for osteogenic differentiation and bone regeneration. Recent findings indicate that these stem cells, such as mesenchymal [13] or induced pluripotent stem cells [14], represent an ideal source for regenerative bone tissue in the dentistry field. 

Although Ca-P-based synthetic materials have long been used as bone substitutes, they are still unsatisfactory. BMP2 has been widely used in bone tissue engineering to improve the osteogenic activity. In this study, we attempted to improve the osteogenic activity using WH bone granules containing BMP2. A successful candidate bone-substitute material should be reproducible, biocompatible, and bioabsorbable [15]. In this study, the WH ceramics, containing Mg ion Ca-P-based bioceramics, were considered to be safe, biocompatible, and effective as a bone substitute for new bone formation [16], because the rate of the WH ceramics regeneration in vivo was faster compared with HAP, and the mechanical strength was much higher. In addition, the Mg ions stimulated cellular proliferation and osteoblast differentiation. Jin et al. reported that the WH material is a more effective bone substrate material for bone treatment in the field of orthopedics [17]. Although Mg ions promote osteoinductivity, WH may still lack sufficient osteoinductivity. Therefore, to overcome the lack of osteoinductivity of WH, we attempted to incorporate BMP2 onto the surface of MP-WH granules. 

In this study, we successfully prepared highly porous WH bone granules using marine plankton. Our results showed that WH was successfully synthesized using marine plankton raw materials via a conversion process involving a hydrothermal reaction. Osteoinduction is an important factor for successful bone regeneration. BMP2 has been widely used to promote osteoinduction. In this study, we incorporated BMP2 protein using an adsorption and lyophilization method [18]. Several studies have reported no correlation between bone formation and the BMP2 dose [19,20]. However, other studies have reported a significant dose-dependent response [21,22]. Uijlenbroek et al. [23] tested several BMP2 doses (1 to 60 µg of BMP for incorporation). It has been reported that bone regeneration increased at a concentration of at least 5 μg or more, and that bone regeneration increased as the BMP2 dose increased, but did not improve ectopic bone formation up to 60 µg. Lin et al. reported that 20 µg of BMP2 was used for coating coralline hydroxyapatite granules and exhibited the highest osteoinductive efficacy [24]. In this study, we chose 20 µg as the optimal dose, and BMP2 incorporation significantly increased osteoblast differentiation compared with the experimental group, which contained only MP-WH granules.

FT-IR spectroscopy and TGA were used for determining the success of the BMP2 incorporation. FT-IR is used for obtaining specific wavelengths that display amide bond peaks to indicate conjugation. Our FT-IR results (Figure 3a) revealed peaks at 1650, 1530, and 1393 cm^−1^, corresponding to the specific peaks of amide I, amide II, and amide III of the protein, respectively. Furthermore, the TGA results (Figure 3b) showed that the weight % decreased as the temperature increased when the organic matter was sintered. These FT-IR and TGA results indicate the successful incorporation of BMP2. Dong et al. reported the BMP protein adsorption onto calcium phosphate ceramic crystals via binding between the functional groups COO−, OH, and NH_2_ of BMP and the calcium site of the ceramic [25]. When BMP2 was adsorbed onto the material, an initial burst of release was observed [26]. This initial burst release may be attributed to the surface-associated proteins. Our release kinetics results showed an initial rapid release pattern up to the first three days, as well as sustained release up to 30 days. The results of this drug release study may have implications regarding early osteoblast differentiation. In addition, the effect of BMP2 can be expected to persist for a long time.

Compressive strength is considered an important factor in bone tissue engineering, because bone graft materials sometimes require sufficient mechanical strength to support loads. In its natural hydrated state, human cancellous bone has compressive strength in the 0.1–16 MPa range [27]. Therefore, a good bone graft material should have sufficient compressive strength for bone regeneration. In the present study, MP-WH prepared from marine plankton exhibited a compressive strength of approximately 20.5 MPa. Porosity is also an important factor for biocompatibility, because it can affect cell ingrowth [28]. Our porosity results suggest 200–500 µm size pore structures, with a porosity of approximately 68% for MP-WH granules. These pore size and porosity values are suitable for bone regeneration. Moreover, these unique interconnected porous network structures provide a larger surface area, which can be utilized for antibiotic loading, the prevention of bacterial micro leakage in the vicinity of the implant-abutment assembly [29], and drug delivery to treat diseases such as mucositis [30] in the dentistry field. 

In our study, the compressive strength and porosity did not significantly change after BMP2 incorporation, with the materials retaining their properties.

The cell proliferation characteristics were not significantly different on day one. This suggests that the BMP2 coating did not significantly affect the adhesion of early cells. However, after five days in culture, the proliferation of cells improved owing to the BMP2 coating. These results show that the growth of mesenchymal stem cells was promoted by the BMP2 coating, and this effect lasted for up to 15 days. Nontoxicity is the most important factor in the development of biomaterials. Our live/dead fluorescence staining results suggest that the prepared MP-WH/BMP2 granules were not cytotoxic.

Cell adhesion on biomaterials is a basic requirement in tissue engineering. Cell adhesion depends on many factors, including extracellular matrix (ECM) proteins and receptors, the composition of the proteins adsorbed to the surface, and the biochemical properties of the surface [31]. Shah et al. reported that BMP2-treated titanium alloys stimulated cell adhesion [32]. Our SEM imaging studies of cell adhesion suggested increased cell adhesion following BMP2 incorporation; this improved cellular adhesion may be attributed to altered cytoskeletal and ECM organization induced by the addition of BMP2 [32]. 

In addition to cellular proliferation and adhesion, the differentiation environment capacity also plays a major role in effective bone regeneration. In this study, we showed that the ALP activity increased in cells interacting with BMP2, suggesting a role for BMP2 in osteogenic differentiation stimulation. Kim et al. reported that BMP2 incorporation induced ALP activity in dental follicle cells during osteogenic differentiation [33]. Similarly, Chung et al. reported that the ALP activity increased in hMSCs using BMP2-incorporating membrane scaffolds [34]. Our ALP activity data agree with these reported results. 

Runx2 is a transcription factor required for osteoblast differentiation [35]. ColIA1, OCN, and OPN also play important roles during osteoblast differentiation, and their expression levels are frequently used as markers for determining the level of osteoblast differentiation [36,37]. We also explored the expression of several osteoblast-related genes and showed that the Runx2, ColIA1, OCN, and OPN expression in hMSCs was stimulated by BMP2 incorporation. Altogether, the ALP activity assay and qRT-PCR analyses indicated that BMP2 incorporation supports an osteoblast differentiation-stimulating environment.

Phosphorylation of Smad 1/5/8 signaling pathways, which directly stimulate osteoblast differentiation, is usually triggered by BMPs [38]. Herein, using Western blotting and immunocytochemistry analysis, we found that BMP2 incorporation markedly increased the phosphorylation of Smad 1/5/8. These results suggest that BMP2-incorporating MP-WH granules promote the BMP2 effect on osteogenic differentiation and trigger Smad 1/5/8 signaling pathways.

**Figure 10:**
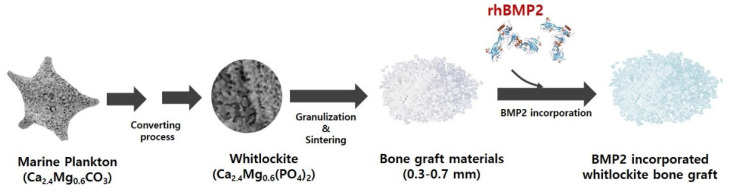
Schematic of the BMP2-incorporating marine-plankton-derived WH bone granules.

In this study, we prepared WH bone graft granules using a marine plankton skeleton, and incorporated BMP2 to further enhance the osteoblast differentiation activity (Figure 10). The prepared MP-WH/BMP2 bone granule material was noncytotoxic. MP-WH/BMP2 enhanced the proliferation and adhesion of hMSCs. In addition, it increased ALP activity and the expression of the osteoblast differentiation marker genes Runx2, Col1A1, OCN, and OPN. These results suggest that marine-plankton-derived WH is a useful bone graft material, and its utility for bone regeneration can be improved by incorporating BMP2. Ca-P-based bone-graft materials have been used in clinical orthopedics and dental surgery for spacing or filling bone defects. The current findings suggest that MP-WH/BMP2 granules are an effective and safe bone-graft material for orthopedics and dental surgery for bone repair.
